# A comparative analysis of two national tuberculosis reporting systems and their impact on tuberculosis case notification in Uganda

**DOI:** 10.4314/ahs.v23i4.3

**Published:** 2023-12

**Authors:** Timothy Kiyemba, Rita Makabayi-Mugabe, Nicholas Sebuliba Kirirabwa, Philip Tumwesigye, Stella Zawedde-Muyanja, Andrew Ocero, Abel Nkolo, Ebony Quinto, Stavia Turyahabwe

**Affiliations:** 1 USAID/Defeat TB project, University Research Co., Kampala, Uganda; 2 The National TB and Leprosy Program, Ministry of Health, Kampala, Uganda; 3 Infectious Diseases Institute (IDI), Makerere University College of Health Sciences, Kampala, Uganda

**Keywords:** web-based reporting, DHIS2, tuberculosis, case notification, Uganda

## Abstract

**Background:**

Before 2018, the use of parallel tuberculosis (TB) reporting systems was resource intensive with duplication of efforts and hence the need to select one that contributed to better TB case notification at the National TB and Leprosy Program (NLTP) in Uganda. We sought to analyse the difference in reporting rates between the two systems in order to improve NTLP TB case notification rates, logistics management, and planning for better health service delivery initiatives.

**Methods:**

We conducted a comparative study to assess TB case notification between the web-based DHIS2 and the district TB supervisor-led health management information system between January 2016 to December 2017. We used Poisson regression analysis to assess the statistical differences in reporting rates between the two reporting systems.

**Results:**

The association between TB case notification and the type of reporting system was statistically significant (Prob > chi2 = 0.0000). The Incident Rate Ratio (IRR) for the web-enabled DHIS2 system versus the district TB supervisor-led health management information system was 1.106625.

**Conclusion:**

The web-based integrated DHIS2 system was more effective in reporting missing TB cases. It presents an opportunity for better planning and allocation of resources for improved service delivery in a low-income setting.

## Background

Globally, since the mid-1990s, the World Health Organization (WHO) supported the implementation of a standardized recording and reporting system for tuberculosis care and control. The paper-based system included tuberculosis (TB) treatment cards, registers, and aggregate reporting formats 1. The advent of information technology and communication (ICT) networks resulted in a gradual shift by many countries from paper-based to electronic information systems, with some countries adopting the aggregate data reporting system and others implemented case-based tracking systems that capture patient-level data 2. This aimed at ensuring that quality and timely data was available for quick decision-making processes. Globally, gaps between the estimated incident TB cases and the number reported are partly due to underreporting of incident cases 3.

Currently, over 70% of countries use aggregate electronic reporting systems for notification of TB cases, with Africa reporting the lowest proportion (54%) 4. More than 40 countries in Africa, Asia, and Latin America have adopted the District Health Information Software Version 2 (DHIS2) as their nationwide Health Information Software including Kenya, Tanzania, Uganda, Rwanda, Ghana, Liberia, and Bangladesh 3. The DHIS2 is an open-source generic web-enabled platform developed and maintained by the University of Oslo for the automation of health and non-health data management and reporting processes by users worldwide 5.

The integrated Ministry of Health (MoH) web-based system powered by the DHIS2 platform was introduced in Uganda in 2011. This was based on the MoH Health Management Information Systems (HMIS) forms and managed by district biostatisticians under the leadership of the MoH Division of Health Information with support from implementing partners. The introduction of this system in Uganda improved the completeness of reporting from 36% in 2011/2012 to 85% in 2012/2013 while the timeliness of outpatient reporting increased from 22% to 78%. Reporting was observed to have improved for other health areas namely immunization where one-year-old children immunized with the three doses of the pentavalent vaccine increased from 57% to 87% 6.

However, for TB case reporting, Uganda implemented the integrated DHIS2-based system alongside a district TB supervisor-led health management system before 2018. This system was managed by District TB and Leprosy Supervisors (DTLS) who visited each of the TB diagnostics and treatment units (DTUs) in their districts to obtain records for TB patients from health facility-based TB unit registers, transferring them into the District TB registers. At the end of each quarter, the District TB registers were used to compile the district quarterly reports which were entered into Microsoft Excel worksheets and sent to the National TB and Leprosy Program (NTLP) by email for consolidation 7.

Implementing both systems concurrently further strained the meager resources available for TB prevention and control. To justify the transition to the integrated MoH DHIS2 for TB reporting recommended by the Ministry of Health resource center, there was no evidence of how its effectiveness in the notification of TB cases in the country compares with that of the traditional DTLS-led system. Quantifying the effect of the system on TB case notification would help the NTLP in Uganda and TB departments of other countries to appreciate the impact of integrated DHIS2 web-based systems in reducing underreporting hence closing the gap of unnotified TB cases.

The US Agency for International Development (USAID) through the Defeat TB project in collaboration with the NTLP sought to analyse the difference in TB case notification as reported by the two systems. The aim was to determine the effectiveness in the notification of TB cases for improved reporting and hence aid logistics planning and aspects of TB health service delivery.

## Methods

### Study population

The study targeted all quarterly TB case notifications between 2016 and 2017 for all 112 districts within Uganda located in East Africa.

### Study design and sampling methodology

The study followed a comparative study design assessing TB case notification as reported by the two different HMIS. The first HMIS is the one capturing data collected by DTLSs and the second HMIS is the one with data compiled by health facility staff with support from health facility records clerks and submitted through the Ministry of Health DHIS2 system. No sampling was done; all TB case notifications for 8 quarters running from January 2016 to December 2017 were considered in this study.

### Data collection and key variable definitions

Secondary data from the two information systems between 2016 and 2017 were combined and analysed to assess for differences. The variable “TB cases notified” referred to incident TB cases registered and reported by the information systems while the variable “system used” was categorized as “1” where the MoH DHIS2 was used and “0” where the parallel NTLP HMIS was used. Other variables used in the study included the period which was categorized into quarters from Jan-Mar 2016 to Oct-Dec 2017 and the 15 regions for Uganda as defined in the MoH DHIS2 8.

### Data management and analysis

The combined dataset assembled under one data structure with the variables system used, TB cases notified, period and region had 1,792 observations for all the quarters based on the quarterly aggregated data reports submitted (Table 1).

**Table 1:** Number of observations for this analysis

Calendar year (quarter)	District quarterly reports submitted through the NTLP parallel HMIS	District quarterly aggregated reports available in the integrated MoH DHIS2	No. of Observations
2016 – Q1	112	112	224
2016 – Q2	112	112	224
2016 – Q3	112	112	224
2016 – Q4	112	112	224
2017 – Q1	112	112	224
2017 – Q2	112	112	224
2017 – Q3	112	112	224
2017 – Q4	112	112	224
No. of Observations	896	896	1,792

*Key: Q1 = Jan-Mar, Q2 = Apr-Jun, Q3 = Jul-Sep and Q4 = Oct-Dec*

This comparative study considered data reported through the MoH DHIS2 as the gold standard based on analysis of data quality assessment (DQA) results for TB case notification to DHIS2 for the urban districts of Kampala, Wakiso, and Mukono which notify 20% of the TB cases in the whole country 9, 10. These showed negligible discrepancies of 1.8%, and 0.9% for the two rounds or assessments conducted by the USAID Defeat TB project 11. Results from the national DQA conducted by the Monitoring and Evaluation Technical Support and Strategic Information Technical Support projects in 2019 also showed a DQA discrepancy of 1.2% between DHIS2 reported and verified figures. These were within the allowable data quality margin of +/-5%.

Deviations were computed by subtracting the figure as reported in the NTLP parallel HMIS from the one as reported in the MoH DHIS2 divided by the figure as reported by the MoH DHIS2. The resulting fraction was multiplied by 100%. A deviation above 10% in magnitude was considered “high”, a deviation between 5% and 10% was considered “moderate” and one below 5% was considered “low”.” A negative deviation pointed to under notification while a positive deviation pointed to over notification of TB cases. These categorizations were in line with those recommended by USAID MEASURE Evaluation 12. Deviations were analysed by quarter and region. Poisson regression analysis was performed to establish if there was a statistically significant difference between TB cases reported by the two systems. Since this was secondary data, there were no particular data cleaning measures undertaken by the study team. However, the respective software had in-built programmed routine data cleaning and validation processes and rules.

## Results

Results from data collected in 2016 showed that a quarterly average of 10,643 incident TB cases were notified using the NTLP parallel HMIS compared to 12,109 reported by the MoH DHIS2. Up to 1,466 incident TB cases reported by the MoH DHIS2 were not reported by the NTLP parallel HMIS (-12.1% discrepancy classified as high since its magnitude was above 10%). The overall total number of incident TB cases notified by the NTLP parallel HMIS during that year (2016) was 42,570 compared to 48,434 reported by the MoH DHIS2. Therefore, the total number of TB cases reported by the MoH DHIS2 but missed notification by the NTLP parallel HMIS was 5,864 (-12.1% discrepancy).

Results from data collected in 2017 showed that a quarterly average of 11,255 incident TB cases were notified using the NTLP parallel HMIS compared to 12,097 reported by the MoH DHIS2. Up to 842 incident TB cases reported by the MoH DHIS2 were not reported by the NTLP parallel HMIS (-7% discrepancy classified as moderate since its magnitude was between 5 and 10%). The overall total number of incident TB cases notified by the NTLP parallel HMIS during that year (2017) was 45,019 compared to 48,386 reported by the MoH DHIS2. Therefore, the total number of incident TB cases reported by the MoH DHIS2 but missed notification by the NTLP parallel HMIS was 3,367 (-7% discrepancy) (Table 2).

**Table 2:** TB case notification as reported in the MoH DHIS2 Vs the parallel NTLP HMIS over time

Period	NTLP parallel HMIS	Integrated MoH DHIS2	TB cases not notified	% Discrepancy	Classification
2016q1	10,522	13,053	2,531	-19.4%	High
2016q2	10,138	12,215	2,077	-17.0%	High
2016q3	11,376	12,282	906	-7.4%	Moderate
2016q4	10,534	10,884	350	-3.2%	Low
**2016 Quarterly** **Average**	**10,643**	**12,109**	**1,466**	**-12.1%**	**High**
**2016 Overall**	**42,570**	**48,434**	**5,864**	**-12.1%**	**High**
2017q1	11,356	12,435	1,079	-8.7%	Moderate
2017q2	11,599	12,489	890	-7.1%	Moderate
2017q3	11,226	12,101	875	-7.2%	Moderate
2017q4	10,838	11,361	523	-4.6%	Moderate
**2017 Quarterly** **Average**	**11,255**	**12,097**	**842**	**-7.0%**	**Moderate**
**2017 Overall**	**45,019**	**48,386**	**3,367**	**-7.0%**	**Moderate**

In 2016, Bukedi region (located in the eastern part of Uganda) reported the highest magnitude deviation in data between the two systems (-30.4%). Bunyoro region reported the lowest discrepancy of -3% while the Teso region was able to notify slightly more TB cases in the NTLP parallel HMIS than those reported in the MoH DHIS2. Generally, all regions apart from Teso reported fewer cases notified to the NTLP parallel HMIS in comparison to TB cases reported in the MoH DHIS2 (Table 3).

**Table 3:** TB case notification as reported in the MoH DHIS2 Vs the parallel HMIS by region

Regions	NTLP Parallel HMIS (2016)	MoH DHIS2 (2016)	% Discrepancy (2016)	NTLP Parallel HMIS (2017)	MoH DHIS2 (2017)	% Discrepancy (2017)
Acholi	2,220	3,028	-26.70%	2,570	2,801	-8.20%
Ankole	2,961	3,433	-13.70%	3,138	3,265	-3.90%
Bugisu	1,556	1,770	-12.10%	1,353	1,792	-24.50%
Bukedi	1,177	1,692	-30.40%	1,183	1,597	-25.90%
Bunyoro	2,271	2,341	-3.00%	2,840	2,759	2.90%
Busoga	3,412	3,490	-2.20%	3,882	4,075	-4.70%
Kampala	6,700	8,217	-18.50%	6,707	7,149	-6.20%
Karamoja	2,099	2,321	-9.60%	2,109	2,291	-7.90%
Kigezi	1,359	1,579	-13.90%	1,409	1,478	-4.70%
Lango	2,989	3,465	-13.70%	2,936	2,938	-0.10%
North Central	3,633	4,415	-17.70%	4,146	4,968	-16.50%
South Central	5,512	5,858	-5.90%	5,363	5,524	-2.90%
Teso	1,120	1,083	3.40%	1,189	1,497	-20.60%
Tooro	2,801	2,892	-3.10%	2,809	2,879	-2.40%
West Nile	2,760	2,850	-3.20%	3,385	3,373	0.40%
**Overall**	**42,570**	**48,434**	**-12.1%**	**45,019**	**48,386**	**-7.0%**

In 2017, Bukedi region reported the highest discrepancy in the TB cases reported to the two systems (-25.9%) while the Tooro region reported the lowest level of underreporting (- 2.4%). West Nile and Bunyoro regions had slightly more cases reported to the NTLP parallel HMIS than the MoH DHIS2. The discrepancies were 0.4% and 2.9% for West Nile and Bunyoro regions, respectively.

The Poisson regression model performed between TB case notification and the system used for reporting was found to be statistically significant (Prob > chi2 = 0.0000). This meant that the system used had a significant effect on TB case notification. The Incident Rate Ratio (IRR) for the DHIS2 system used in comparison to the parallel NTLP HMIS was 1.106625 meaning that the TB cases notified using the integrated MoH DHIS2 were 10.6% higher than the cases notified using the parallel NTLP HMIS (Table 4).

**Table 4:** Poisson regression analysis between TB case notification and system used for reporting

Item	Overall values	2016 values	2017 values
Incident Rate Ratio (IRR)	1.106625	1.140295	1.074791
P-value	0.000	0.000	0.000
Standard error	0.0051604	0.0075757	0.007038
95% confidence interval			
Lower bound	1.096557	1.125543	1.061085
Upper bound	1.116786	1.155240	1.088674

For 2016, the IRR for the DHIS2 system compared to the NTLP HMIS was 1.140295 indicating that the TB cases notified using the DHIS2 system were 14% higher than those notified using the parallel NTLP HMIS while in 2017, the IRR for the DHIS2 system compared to the NTLP HMIS was 1.074791 indicating that the TB cases notified using the DHIS2 were 7.5% higher than those notified using the parallel NTLP HMIS.

## Discussion

We carried out a comparative study that showed that the web-enabled integrated MoH DHIS2 was more effective in reporting missing TB cases in comparison to the parallel NTLP HMIS. The lower TB case notification rates in the parallel NTLP HMIS could be attributed to the fact that it was difficult for DTLSs to reach every health facility, leading to several sites missing out on having their reports included in the system 13.

The system was supported mainly by NTLP and a few TB partners in the country with minimal support from the MoH Division of Health Information and other stakeholders. The higher TB case notification observed using the web-based MoH DHIS2 system indicates that a significant proportion of TB cases notified were missed and not notified by the NTLP parallel HMIS. The MoH DHIS2 system provided a better opportunity for more complete reporting 6, 14 and higher TB case notification to facilitate projections for key logistics and resources necessary to support incident TB cases. The introduction of web-enabled systems in low-income countries has enabled online interaction and viewing of results by a wide range of stakeholders with convenience ensuring greater transparency and monitoring compared to standalone non-web-enabled systems. Data from multiple centers can easily be collated and reports generated rapidly 15.

The qualitative experience in using DHIS2 in Bangladesh was cited to have improved the timeliness and completeness of reporting 14. The adoption of DHIS2 in Kenya in 2011 sustainably improved the completeness of data for most key malaria indicators 16. The introduction of DHIS2 in Uganda led to improved timeliness and completeness of reporting and subsequently the quality of data reported for outpatient and inpatient health service usage 17.

Using web-based reporting platforms like DHIS2 for collecting TB data at the health facility or other lower levels in the community should be encouraged to ensure the completeness of the data reported. This also reduces the data collection burden of health workers and saves costs on transport to collect the reports manually from sites. Web-based platforms also ensure data is available to a wide range of stakeholders and quality is ensured for improved reporting and decision making.

The study's strength lies in the fact that this was national data that involved large datasets and hence findings provided a national perspective to paper-based reporting versus web-based reporting and its impact on TB case notification. However, the limitation was that information on timeliness and completeness was not available for the DTLS-led system to allow for a comparison between the two approaches. This can be considered for future research through primary data collection. Data to facilitate analysis for this study was readily available in the MoH DHIS2 and in excel files at the NTLP however, comparable data was for only two years (8 quarters) because more consistent data points for TB case notification were available starting in 2016 while reporting using the DTLS system stopped by end of 2017. The availability of more data points would have strengthened the validity of the results. Future research should consider more data points to strengthen the findings.

## Conclusion

Working with a web-enabled, nationally institutionalized HMIS for managing TB data allows for broader engagement of national HMIS stakeholders enabling leveraging of resources and support for TB data management at all levels of the health system. The DHIS2 platform makes it easier for health facilities to report data and for national, regional, and district teams to validate it leading to improved quality and completeness of reported data. This, in turn, enhances the quality of monitoring, action planning, and decision-making by NTLP and other users of the routinely collected TB HMIS data.

Using the web-based MoH DHIS2 contributed to an increase in the number of TB cases notified to the NTLP, and a decrease in the number of documented missed TB cases. Accurate data will help improve planning and resource estimates for TB programming, providing more patients with access to care and treatment. Therefore, to decrease the disparity of unreported TB cases, TB stakeholders in Uganda and other developing countries should actively promote the utilization of this system for reporting TB cases and other relevant health information.

## Figures and Tables

**Figure 1:**
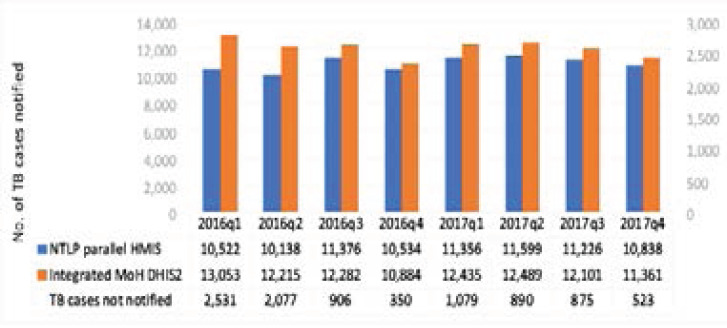
Graph showing comparative analysis for TB cases notified by the two systems

## Data Availability

The datasets used and/or analysed during the current study are publicly available by reasonable request to the corresponding author, whose email is available in this submission.

## List of abbreviations

CI: Confidence Interval
DHIS2: District Health Information Software Version 2
HIV: Human Immunodeficiency Virus
HMIS: Health Management Information System
IDI: Infectious Diseases Institute
IRR: Incident Rate Ratio
MoH: Ministry of Health
NTLP: National TB and Leprosy Program
TB: Tuberculosis
URC: University Research Company
USAID: United States Agency for International Development
WHO: World Health Organization
